# Supplementary material for: Are carbon emissions trading and green financial instruments synergistic? -Comprehensive quantitative research based on content analysis
Source: PLoS One. 2024 Mar 7;19(3):e0298601. doi: 10.1371/journal.pone.0298601 (PMC10919601; doi:10.1371/journal.pone.0298601)
Supplement: S1 Data — (ZIP) [file pone.0298601.s001.zip › Data/地级市Z_F工作报告.zip!/╕ú╜¿/╕ú╓▌╩╨/└┤╫╘╣1⁄2╓┌║┼í╛┬φ┐╦╩2╛▌═°í┐_2015.docx]

各位代表:

　　现在,我代表福州市人民政府向大会作政府工作报告,请各位代表审议,并请市政协各位委员和其他列席人员提出意见。

　　一、2014年工作回顾

　　2014年,在省委、省政府和市委的正确领导下,我市各级政府认真贯彻落实党的十八大、十八届三中、四中全会精神,深入学习贯彻习近平总书记系列重要讲话和来闽考察重要讲话精神,牢牢把握中央支持福建加快发展的重大战略机遇,全力以赴稳增长、调结构、促改革、惠民生,较好地完成了市十四届人大三次会议确定的各项任务。一年来的主要工作及成效是:

　　(一)经济持续稳步增长。预计全市生产总值突破5000亿元,增长10.5%左右,经济总量迈上新台阶;一般公共预算总收入780.48亿元,增长13.3%,一般公共预算收入510.87亿元,增长12.5%,均提前一年完成“十二五”规划任务;固定资产投资(不含平潭)4128亿元,增长18%;出口总额205亿美元,增长6%;按验资口径实际利用外资15.47亿美元,增长8.1%;社会消费品零售总额2982.1亿元,增长14.2%。实施480项重点项目建设,完成投资1750亿元,为年度计划的109.3%。

　　(二)产业优化升级步伐加快。全市规模以上工业增加值1831亿元,增长12%。产业布局加速向南北“两翼”拓展,“两翼”地区工业总产值占全市比重达54%。完成工业投资1175亿元,增长13.4%,天辰耀隆己内酰胺等63个工业重点项目建成投产。现代服务业提质发展,海西现代金融中心区获批设立,一批城市综合体建成开业,成功举办69场大型展会,全市接待游客3996万人次,福州被国家列为海峡两岸电子商务试验区、电子商务与物流快递协同发展试点城市。成功开辟福州至纽约空中航线,福州航空公司正式成立。实现高新技术产业产值3400亿元,新增省级以上创新型企业29家,新认定市级以上企业技术中心21家、院士(专家)工作站32家,国家半导体照明国际创新园落户福州。闽都人才聚集工程与中国福州海西引智试验区建设深入推进,人才工作体制机制创新和政策扶持力度加大,福州与北京师范大学等10所高校签署全面合作协议,新引进省“百人计划”以上高层次人才14人、人才团队9个。

　　(三)改革创新活力增强。国企改革不断深化,16个市直部门与所管辖的107家企业完全脱钩。首批推出73项总规模853亿元的项目吸引民间投资,PPP试点等新型投融资模式有效运用。城区道路清扫保洁、道路绿地养护全部实行市场化运作。福州成为全省保留市级行政许可事项最少的城市,并率先实施全国首部市级行政服务地方性法规。市行政服务中心荣获中国“质量之光”质监改革创新示范奖,市民服务中心基本建成。政府机构改革基本完成,事业单位分类改革稳步实施。财税、教育、文化、卫生、户籍等制度改革扎实推进。农村综合改革向纵深拓展,土地股份合作经营、林权流转等试点工作有效开展。

　　(四)大开放格局加速形成。福建自贸区福州片区获国家批准设立。主动融入国家“一带一路”战略,成功举办21世纪海上丝绸之路市长(高峰)论坛、首届丝绸之路国际电影节等一批重大活动,中国—东盟海产品交易所上线试运营,与印尼等东盟国家合作新建境外综合渔业基地、养殖基地初具成效。罗源湾口岸扩大开放获批。新签约对接“三维”项目413项、总投资3618亿元,履约率79%;新批千万美元以上外(台)资项目29项。成功举办“5·18”海交会、海峡青年节、海峡两岸合唱节、海峡渔业周·渔博会等对台特色交流活动,引进台资金融机构等工作实现新突破。积极支持平潭开放开发,渔平高速公路延伸线等项目建成。福莆宁同城化步伐加快,对口支援和山海协作工作有效开展。榕港榕澳合作和侨务、外事、异地商会等工作取得新成果。

　　(五)福州新区开发建设取得突破。基本编制完成福州新区发展规划、空间发展规划纲要、水资源论证和环境影响评价。三江口、琅岐岛以及长乐航空新城、福清海港新城等重点区域建设扎实推进,新区300项重点项目完成投资约1100亿元,为年度计划的110%。新开工建设滨海大通道、东部快速通道和江阴港8号、9号码头泊位等一批项目,建成福清核电1号机组、江阴港铁路支线等一批项目。

　　(六)宜居城市建设成效显著。新改扩建城市道路80公里,完成城区路网7个节点改造。在全省率先推行定制公交车,新增更新公交车445辆、出租车1677辆,新辟优化公交线路131条。地铁1号线8个区间实现双线贯通,2号线启动建设。实施三环路绿化等重大绿化项目和20座跨江大桥、高架桥、人行天桥的花化彩化,新改扩建16个公园,建成绿道与慢行系统101.3公里,新增城市园林绿地280万平方米。内河综合整治持续推进,江北城区70%河道实现去黑除臭,完成左海清淤工程。道路交通安全综合整治、老旧住宅小区综合整治、“两违”治理等专项整治扎实开展,数字城管系统实现五城区全覆盖。节能减排攻坚力度加大,创建国家生态市、国家森林城市活动深入开展,全市空气质量位居全国74个重点城市前列,市级集中式饮用水源地水质达标率为100%,福州经济技术开发区通过国家生态工业示范园区考核验收,4个县(市)区和30个乡镇通过国家级生态县(市)区、生态乡镇考核验收,福州被评为全国首批创建生态文明典范城市。

　　(七)城乡一体发展协调推进。全市农林牧渔业总产值713亿元,增长4.3%,产值亿元以上农业产业化龙头企业突破百家,福州茉莉花与茶文化系统入选全球重要农业文化遗产。实施21个重大水利项目建设,综合治理水土流失面积13.86万亩。持续改善农村生产生活条件,新建改造农村公路222公里,新建农村户用沼气池1000口,新建改建农村无害化卫生户厕2050户,新建空白村卫生所71个,新解决41万农村人口饮水安全问题,完成“造福工程”搬迁5020人。省、市试点小城镇建设扎实推进,一批美丽乡村精品示范村建设成效明显,闽侯孔元村入选中国最美休闲乡村。

　　(八)各项社会事业全面发展。新改扩建10所中小学、10所公办幼儿园,新增中小学学位5450个、学前教育学位3150个,均衡教育、在线德育、体艺美育、幸福教育等工作走在全省前列。医疗卫生服务水平不断提升,建成肺科医院负压病房楼等项目,组建精神卫生医疗联合体。县级公立医院综合改革全面启动。公共文化服务不断拓展,文化惠民“六进”活动持续深化,首批24个街区24小时自助图书馆建成投入使用,闽剧《兰花赋》晋京展演取得圆满成功。茉莉花茶窨制工艺、咏春拳等入选国家级非遗代表性项目名录。首届全国青运会筹备工作有序推进,海峡奥体中心、运动员村以及各新建、改造比赛场馆基本建成。成功举办世界杯龙舟赛、中国羽毛球公开赛、环福州·永泰国际公路自行车赛等一批大型体育赛事,我市运动员在亚运会、青奥会上取得优异成绩,在省运会上获金牌数、团体总分、破纪录数三项第一。计生服务水平稳步提高,“单独两孩”政策稳妥实施,全市人口自然增长率控制在9.3‰以内。社会福利、社会救助、社会慈善等工作有效开展,居家养老服务工作入选“中国社会治理创新范例”。民族宗教、新闻出版、广播影视、社会科学、地方志、妇女儿童、老龄、残疾人等事业加快发展。双拥共建、国防动员、民兵预备役建设、海防、人防等工作扎实推进。

　　(九)民生工作不断加强。全市各级财政用于民生支出452.27亿元,占一般公共预算支出的79.2%。年初确定的市级25件71项为民办实事项目件件有落实。城镇新增就业14.54万人,转移农业富余劳动力5.04万人,城镇登记失业率2.42%,城镇居民人均可支配收入32390元,增长9.2%,农村居民人均可支配收入14020元,增长11.2%。城乡基本养老保险制度实现全覆盖,城乡低保、农村“五保”和城乡居民医保财政补助标准进一步提高。基本建成保障性住房1.18万套,配租配售等制度不断完善。市场物价保持稳定,居民消费价格总水平上涨1.8%,主要食品安全检测指标总体达标。“平安福州”建设成效明显,公众安全感达92.72%,生产安全事故发生起数和死亡人数分别下降7%、18.8%。信访、行政复议、法律援助、社区矫正、青少年事务社工等工作不断加强。

　　(十)政府工作作风切实改进。党的群众路线教育实践活动和“四个万家”等活动深入开展,各级领导干部深入基层、企业约86万人次,全市会议、文件简报数量和“三公”经费支出分别下降23.7%、17.8%和29%,市级考核检查项目压缩80%。机关效能建设向纵深拓展,媒体直播民主评议等活动受到群众好评。政府信息化建设水平不断提升,在全国主要城市电子政务发展排名中福州位居第5位。行政监察、审计监督等工作力度持续加大。认真执行人大及其常委会决定决议,自觉接受人大及其常委会的法律监督、工作监督和政协的民主监督,大力支持政协开展协商民主活动。提请市人大常委会审议地方性法规草案6件,制定政府规章和规范性文件23件。办复531件省、市人大代表建议和562件省、市政协提案,满意率分别为98.2%和99.8%,办结率均为100%。

　　各位代表,回顾过去一年的工作,我们取得的成绩来之不易。这是在省委、省政府和市委正确领导下,全市人民团结拼搏的结果。在此,我代表市人民政府,向全市人民致以崇高的敬意!向人大代表、政协委员、各民主党派、工商联、各人民团体、无党派人士、离退休老同志及社会各界人士,向中直、省直机关企事业单位和驻榕部队、武警官兵、公安政法干警,向关心支持福州发展的港澳台同胞、海外侨胞、国际友人,表示衷心的感谢!

　　在看到成绩的同时,我们也必须正视当前福州发展中存在的困难和问题,主要是:经济总量不够大、产业结构不够优、创新驱动能力不够强,缺乏产业大项目和战略性新兴产业支撑;空间、资源、环境刚性约束趋紧,环境保护和生态建设任务较为艰巨;城市规划建设管理水平有待进一步提升,停车难、交通拥堵现象亟需缓解,地铁等市政项目建设进度滞后;社会保障水平还不够高,教育、医疗、文化等资源均衡化问题需要加快解决;一些干部服务意识不强,仍然存在推诿扯皮、本位主义、“庸懒散拖”以及不作为、乱作为等问题。对此,我们将切实采取有效措施,认真加以解决。　二、2015年工作安排

　　各位代表,今年是全面深化改革的关键之年,是全面推进依法治市的开局之年,也是全面完成“十二五”规划的收官之年和推动福州科学发展跨越发展的重要一年。根据市委统一部署,今年政府工作总体要求是:全面贯彻落实党的十八大和十八届三中、四中全会精神,深入学习领会习近平总书记系列重要讲话和来闽考察重要讲话精神,主动适应经济发展新常态,抢抓中央支持福建加快发展的重大战略机遇,坚持稳中求进,以提高经济发展质量和效益为中心,以加速推进福州新区开放开发、自贸区建设和打造21世纪海上丝绸之路战略枢纽城市为重要平台,深化改革开放,强化创新驱动,加快转型升级,持续改善民生,推进依法治市,在更高起点上加快建设闽江口金三角经济圈,努力为建设机制活、产业优、百姓富、生态美的新福建做出新贡献。

　　今年全市经济社会发展主要预期目标是:地区生产总值增长10.5%左右;一般公共预算收入增长10.5%左右;固定资产投资增长18%左右;出口总额增长6%;按验资口径实际利用外资增长6%;社会消费品零售总额增长13%;居民消费价格总水平涨幅控制在3%左右;城镇居民人均可支配收入增长9.5%,农村居民人均可支配收入增长10.5%;城镇登记失业率控制在4%以内;人口自然增长率控制在9.5‰以内;完成单位生产总值能耗、化学需氧量、二氧化硫、氨氮、氮氧化物等年度节能减排任务。

　　今年大事多,做好各项工作意义重大、责任重大。我们要紧紧抓住中央支持福建加快发展这一重大战略机遇,确保完成“十二五”规划,全面推动福州科学发展跨越发展;我们要加速推进福州新区开放开发,扎实抓好自贸区建设,加快打造21世纪海上丝绸之路战略枢纽城市,增创福州发展新优势;我们要当好东道主,精心办好首届全国青运会;我们要全力创建国家生态市,为全省建设生态文明先行示范区做出省会城市更大的贡献。

　　为实现上述预期目标和主要任务,重点抓好八个方面工作:

　　(一)大力实施福州科学发展跨越发展行动计划。

　　科学编制“十三五”规划。突出福州特色,加强经济转型升级和优化产业布局、基础设施布局、城市布局、区域布局等重大战略问题的研究。科学制定全市“十三五”发展规划纲要,明确福州未来发展的思路、目标和任务。认真编制各专项规划,形成协调衔接、成龙配套的规划体系。深化重大项目规划与论证,积极生成一批关系发展全局、关系民生的重大项目。

　　全面推进福州新区开放开发。认真做好福州新区申报国家级新区各项工作,争取福州新区开放开发尽快上升为国家战略。加快新区总体规划以及综合交通等专项规划编制,推进新区“多规合一”前期规划研究。积极创新新区运行机制,实施“小政府、大服务”的大部制、扁平化管理。大力加快北部、中部、南部三大片区建设,形成新区大开发、大建设格局,争取完成固定资产投资2000亿元以上。全面推进环南台岛滨江休闲路、滨海大通道、东部快速通道、马尾大桥、琅岐环岛路等一批重大项目建设,动工建设道庆洲大桥、海峡文化艺术中心等一批重大项目。

　　认真实施项目带动战略。实行项目分级管理、分类推进,提高项目开工率、投产率,初步安排市级重点项目598项,年度计划投资1982亿元。坚持招大商、招好商,强化“三维”对接和专业化招商,形成“签约一批、开工一批、投产一批”的滚动发展态势。坚决突破项目征地拆迁瓶颈制约,加快解决重大项目交地和历史遗留的逾期交地问题,全力保障项目用地。加强政银企对接,创新融资方式和渠道,强化重大项目建设资金保障,积极争取委托省里发行地方债券,扎实做好PPP试点等工作。着力保障重点项目用砂需求,推广应用机制砂,建设一批机制砂生产基地。

　　大力强化基础设施支撑。积极推进在建的福平铁路等4条铁路建设,建成合福铁路福州段、可门港铁路支线、福州东(樟林)货车车辆段,争取动工建设松下港铁路支线,抓好福(州)马(尾)铁路改造等前期工作。加快在建的绕城公路东南段等4条高速公路建设,建成京台高速建瓯至闽侯段、沈海复线宁德至连江段。争取动工建设江涵大桥,加快外郊快速环线新规划路段等项目前期工作。进一步加快福州海港、空港发展,推进江阴、罗源湾、松下等港区大型深水码头泊位以及长乐机场二期建设,争取福州港口年货物吞吐量突破1.2亿吨、长乐机场年旅客吞吐量突破1000万人次。积极推进福清核电2号~6号机组、永泰抽水蓄能电站、神华煤港电一体化等能源项目建设。加快闽江下游南港防洪、葫芦门水库、霍口水库等水利工程建设,动工建设江北城区山洪防治工程。

　　(二)全力开创改革开放新局面。

　　持续深化市场导向的各项改革。全面完成新一轮政府机构改革,深入推进事业单位分类改革。进一步提升市行政服务中心服务水平,建立行政审批项目动态清理机制,市民服务中心建成并投入使用。强化国有资产经营管理,实施四家投资集团权属企业的改制重组和整合。大力支持非公经济健康发展,在项目投资、融资服务、财税政策、土地使用等方面一视同仁,鼓励和引导民间资本以多种形式进入基础产业、社会事业、特许经营等领域。继续推行政府购买服务,实施垃圾收运、内河整治管养等领域的市场化运作。积极推进不动产统一登记。加快农村土地承包经营权和集体建设用地使用权、宅基地使用权、农村房屋所有权、林权等确权登记发证,稳妥推进流转、抵押等机制建设。全面推行市、县两级全口径预算管理。

　　大力推进自贸区建设。立足改革创新,坚持先行先试,高起点、高水平谋划和推进自贸区建设发展,加快信息化平台等建设,率先推行各类投资便利化、贸易自由化政策。凸显对台和海洋特色优势,争取在两岸交流交往中走在前列,重点建设先进制造业基地、“海丝”建设重要平台、两岸服务贸易与金融创新合作示范区。完善自贸区功能规划和产业布局,加强投资管理体制、贸易监管、金融服务等方面创新,全力打造符合国际惯例的良好营商环境,加快形成具有福州特色的自贸区运作模式。

　　积极打造21世纪海上丝绸之路战略枢纽城市。拓展与“海丝”沿线国家和地区在投资、贸易、基础设施、技术等领域的合作,做大做强中国—东盟海产品交易所,加快建设毛里塔尼亚综合渔业基地二期、几内亚比绍综合渔业基地和印尼、缅甸养殖基地等项目。进一步办好丝绸之路国际电影节、亚洲合作对话——共建“一带一路”工商大会等活动,积极申办21世纪海上丝绸之路博览会、海上丝绸之路国际旅游节,推动“海上丝绸之路:福州史迹”申报世界文化遗产,打造一批“海丝”人文交流品牌。加强电子信息产品、船舶、家具及装饰品、钟表等重点出口商品基地建设,主动对接中韩、中澳等自由贸易协定,巩固传统国际市场,积极开拓出口新兴市场。鼓励有条件的企业“走出去”发展。进一步维护侨益、发挥侨力,强化与海内外重点客商以及华侨华人社团的联系,做好新华侨华人、华裔新生代工作,更加积极地引导海内外乡亲“回归”发展。争取外国领馆入驻福州。

　　不断加强对台交流合作与区域协作。进一步推进榕台在先进制造业、现代农业以及金融、电子商务等产业的深度对接合作,加快台商投资区、台湾农民创业园等园区发展,积极打造海峡两岸电子商务经济合作试点城市。开通黄岐至马祖客运航线,加快构建环马祖澳旅游区。加强台胞协调服务和权益保障平台建设。深化榕台科技、教育、文化、卫生、体育以及宗教文化、“祖地文化”等领域的全方位交流,办好“5·18”海交会、海峡青年节、海峡两岸合唱节、海峡渔业周·渔博会等一批对台特色交流活动,规划建设海峡青年交流基地,加快建设闽台(福州)文化产业园。不断深化与港澳在金融、物流、旅游、文化创意等领域的务实合作。加强泛珠三角、闽浙赣皖、闽东北区域协作、山海协作,办好泛珠省会城市市长联席会议等活动,继续抓好对口援疆援藏援宁工作。加快福莆宁在交通建设、产业发展、公共服务等领域的同城化步伐。

　　扎实推动与平潭联动发展。谋划推进福建自贸区福州片区与平潭片区的互动联动和差异化发展。主动争取平潭综合实验区政策辐射,促进平潭与周边地区有机联动发展。大力支持平潭重大项目建设,积极推进闽江北水南调(平潭引水)、长平高速公路等项目建设。继续在科技、教育、卫生、社保、人才培训、干部交流等方面为平潭提供良好服务,实现医保管理服务同城化。(三)加快构建经济升级版。

　　提速发展都市特色现代农业。认真实施“粮安工程”,确保粮食种植面积稳定在160万亩,力争完成22万吨现代化仓容建设任务。大力发展特色优势农业和品牌农业,进一步打响福州茉莉花茶、金鱼、鱼丸等品牌,推进茉莉花茶产业园、金鱼产业园等一批园区建设。积极发展设施农业、生态农业、休闲观光农业和林下经济,加强农田水利基础设施建设,新增设施农业3000亩。引导和规范土地有序流转,发展壮大一批农业龙头企业,加快培育一批专业大户、农民专业合作社、家庭农场等新型农业经营主体。扶持品种改良和良种引进,培育一批适合本地种植的良种。筹建市农业科学研究院,完善农业科技推广和服务保障体系。

　　努力壮大先进制造业。实施产业龙头促进计划,抓好申远新材料、美得石化、巴陵石化、福清触控面板、马尾船政(连江)特种船舶、科立视二期等150个项目建设,建成中石油渤海装备福建钢管项目一期、中景石化、中江石化、景丰科技等60个项目,争取新增120家规模以上企业,新增1个千亿产业集群、5家百亿企业。加快新设立园区基础设施建设,积极引导现有工业园区向专业化、特色化、规模化方向发展,抓好软件园五期等项目建设,着力打造一批千亿产业园区。全面加强机械、纺织、冶金、建陶等传统产业的技术改造,实施100项重点技改项目。鼓励企业通过上市等方式实现增资扩产、发展壮大。大力实施商标、品牌和质量强市战略,支持企业收购国际知名品牌,加快培育一批名牌产品。

　　切实提升服务业发展质量。抓好鼓楼区国家服务业综合改革试点,打造“两江四岸”等现代服务业聚集区,培育壮大总部经济、楼宇经济。不断拓展商贸、家庭和健康服务等生活性服务业,推进大东街口商圈改造提升,加快台江苏宁广场二期、万宝商圈地下人防工程等一批城市综合体建设。结合市区夜市摊点整治,积极打造一批各具特色的“美食一条街”、美食园。鼓励和引导各县(市)加快城关服务业提升步伐。提速发展冷链物流、绿色物流、电子商务物流等现代物流业。大力发展电子商务,推进国家电子商务示范城市建设,加快阿里巴巴“一达通”项目运营和京东电商园、海峡电子商务产业基地二期等项目建设。着力申报服务外包示范城市。加快建设海西现代金融中心区,大力引进一批有影响力的金融机构,支持发展基金、小额贷款公司、风险投资公司、股权投资公司,鼓励开展资产证券化等金融创新。抓好贵安、桂湖、龙祥岛、东壁岛、三江口、云顶等一批大型旅游项目建设与运营,推进“三坊七巷”创建国家5A级旅游景区、鼓岭创建国家级旅游度假区,打造国内外知名的养生休闲度假旅游城市。办好中国国际体育用品博览会等一批大型展会。发展壮大福州航空公司,不断扩大航线覆盖面。

　　积极培育高新技术产业和战略性新兴产业。大力发展互联网经济,推进物联网、云计算、大数据等产业发展,加快数字福建(长乐)产业园等项目建设。积极发展轨道交通制造、通用航空制造、智能装备制造等产业,启动建设长乐MS760飞机制造、中航国际通用航空飞机制造等项目。继续支持生物与新医药、新材料、新能源、节能环保等产业发展。鼓励企业加大研发投入,争取新增省级以上创新型企业20家,新认定市级以上企业技术中心、工业设计中心20家,支持办好中科院海西研究院等一批创新机构,力争全市高新技术产业产值突破3800亿元。加大知识产权运用和保护力度。发挥中国福州海西引智试验区政策优势,大力实施闽都人才聚集工程,加强与国内外知名高校、科研院所、榕籍院士的合作,办好海峡两岸(福州)大学生创业创新大赛等活动。

　　不断做大做强海洋经济。围绕推进“海上福州”建设,加快闽台(福州)蓝色经济产业园、海峡现代渔业经济区远洋渔业加工区等项目建设,发展壮大八大“蓝色产业”。积极开发海洋牧场,探索陆上工厂化海水养殖,支持罗源、连江稳妥有序推进罗源湾养殖退养和渔民转产转业。大力发展远洋捕捞和海产品精深加工。科学保护和开发海域、海岸、海岛资源,开展市级海洋功能区划、无居民海岛保护和利用规划修编。支持福州港整合资源、开辟航线,加强与内陆地区在港口方面的合作。

　　(四)持续推进新型城镇化和城乡融合发展。

　　着力增强中心城市承载服务功能。加快中心城区路网、过江桥梁等基础设施建设,新改扩建城市道路80公里、燃气管网70公里、市政供水管网140公里,建成海峡奥体中心片区配套道路等新建项目和福湾路、金鸡山隧道、金山大桥复桥、化工路、远洋路等提升改造项目。新增更新公交车350辆,新辟优化公交线路20条,改造公交候车亭100个,投放“预约出租车”500辆,继续推广公共便民自行车。新增公共停车泊位1000个以上,每个城区至少新建1个立体停车库。全面提速地铁建设,实现1号线全线贯通,加快2号线建设,力争启动6号线建设。

　　努力实现城市环境面貌明显改观。加强海峡奥体中心、火车北站、火车南站等片区环境整治,推进“三边三节点”和“四线”可视范围景观提升。启动实施旧屋区改造473万平方米。综合整治100个以上老旧住宅小区,进一步规范住宅小区物业管理。全面完成飞凤河、台屿河等6条内河整治,启动清富河、流花溪等7条内河整治。新改扩建飞凤山奥体公园、南公园、江心公园等10个公园,新建温泉公园—金鸡山公园生态廊道、左海—金牛山森林步道等城市绿道和慢行系统30公里,精心打造建新大道等3条亮丽景观大道,提升闽江两岸等灯光夜景工程。实施绿荫行动计划,加大榕树在园林绿化上的应用。推进建筑工业化生产和绿色建筑发展。全面拓展“智慧城市”管理服务平台应用,持续治理“两违”、非法采砂、渣土车违规、交通拥堵等突出问题,提升城市网格化、数字化、精细化管理水平。

　　扎实推进新型城镇化和美丽乡村建设。鼓励各县(市)因地制宜发展壮大特色产业,做大做强县域经济。积极实施“大城关”战略,促进县(市)城关扩容提质、更新改造。加快新型城镇化规划编制,开展“多规合一”试点,加强省、市试点小城镇建设和青口、江阴“小城市”培育试点。深化户籍制度改革,推行居住证制度。推进各具特色的美丽乡村建设,争取建成130个美丽乡村、12个美丽乡村精品示范村。组织实施“新农村幸福家园工程”,按照“十个一”标准建设9个示范村。加大科学扶贫、精准扶贫力度,深化县(市)区对口协作,继续帮扶闽清、永泰等欠发达县和老少边贫岛地区发展。深入开展“春风·春雨·光彩”、“榕商联村”等扶贫济困活动,实施“造福工程”搬迁4000人。

　　(五)积极建设生态文明先行示范区。

　　完善生态文明制度。加快环境总体规划编制,科学划定生态保护红线,严格落实环保监管“一岗双责”和分级管控制度。完善生态补偿和资源有偿使用制度,推进水权、排污权有偿使用和交易试点,探索开展碳排放权交易。造林绿化9.7万亩,扩大重点生态区位非国有商品林赎买试点。完成国家生态市、省级森林城市创建任务,深入开展创建国家级生态县(市)区、生态乡镇等活动,推广绿色消费、低碳出行的生活方式,积极开展低碳社区试点工作,办好中国生态文明论坛福州年会。加强生态环境法治建设,健全企业环保失信追责和环境损害赔偿制度,严厉打击各类环境违法行为。

　　推进节能减排攻坚。加强源头管控,实行能耗强度、碳排放强度和能源消费总量控制。实施30项重点节能项目、300项重点减排项目,支持闽清加快完成建陶业整体煤改天然气工程。积极发展绿色经济、循环经济、低碳经济,持续开展生活垃圾分类等循环利用试点。完善环保基础设施,推进红庙岭垃圾焚烧发电厂二期、洋里污水处理厂四期以及地下垃圾转运站等垃圾、污水处理设施建设,新改建污水管网130公里以上。

　　强化环境重点治理。实施大气污染监督网格化管理,坚决治理工业废气、机动车尾气、施工和道路扬尘、垃圾焚烧、餐饮油烟等污染,保持空气质量位居全国74个重点城市前列,努力实现“保十争五”的目标。深入开展闽江、敖江、龙江、大樟溪等重点流域水环境综合整治,加强畜禽养殖、石板材等重点行业污染治理。强化对重金属、危险化学品、核设施和放射源安全监管,提高突发环境事件应急处置能力。继续治理“青山挂白”、乱建坟墓,推进闽江河口湿地等自然保护区生态保护与修复,加强水土资源流失治理和节约集约利用。

　　(六)统筹协调发展各项社会事业。

　　办好首届全国青运会。精心组织青运会开闭幕式、赛事活动和市场化运作,认真做好安保、后勤保障、志愿服务等工作。大力推动“全民健身与青运同行”,切实办好中国羽毛球公开赛、国际沙滩排球巡回赛、环福州·永泰国际公路自行车赛、全球华人篮球邀请赛等一批大型赛事,新建15个城市社区多功能运动场、250条健身路径,继续打造以健身步道和绿道为主的10分钟健身圈,掀起全社会关心支持青运会、推动全民健身的热潮。

　　促进教育优质发展。加快学校基本建设,新改扩建10所中小学、10所公办幼儿园,新增中小学学位5000个、学前教育学位2000个,采取政府购买服务方式扩大普惠性幼儿园覆盖面,加快解决被拆迁中小学回迁安置问题。扎实推进素质教育,深入践行幸福教育。健全城区义务教育招生机制,完善小升初招生、中招政策,加大教育资源配置向农村特别是边远、贫困、民族乡村倾斜力度,力争实现全市义务教育县域内基本均衡发展。推动4所普通高中达标升级。提升高校建设内涵。深化校企融合,推进中高职一体化发展,完善现代职业教育体系。规范、拓展学生托管工作。提高师德水平和业务能力,启动实施学前教育师资队伍学历提升五年计划。规范发展民办教育。扶持发展特殊教育、终身教育,加快建设学习型城市。

　　提高医疗卫生服务水平。深化医药卫生体制改革,全面推进县级公立医院综合改革,启动城市公立医院综合改革。进一步增加医疗资源,动建市妇幼保健院新院等项目。完善公共卫生服务体系,提高疾病预防控制、卫生应急和卫生监督执法能力。加快组建中医、肝病等医疗联合体,促进优质医疗资源共享。改造提升16家乡镇卫生院、社区卫生服务中心和56个村卫生所,加强基层医疗卫生人才队伍、医疗用房建设和设备配备。强化临床重点专科建设。逐步推行医师多点执业。支持和规范社会资本办医。大力扶持中医药事业发展。加大医患纠纷调处力度。广泛开展爱国卫生运动。进一步提高基层计划生育服务能力,促进人口长期均衡发展。

　　增强城市文化软实力。深化市歌舞剧院等国有文艺院团体制机制改革,加快国有经营性文化单位转企改制。继续实施文化惠民工程,推进第二批26个街区24小时自助图书馆等项目建设,建成海峡图书馆、海峡妇女儿童活动中心等一批重大项目,申报国家公共文化服务体系示范区。切实加强历史文化名城保护,加快朱紫坊、上下杭、烟台山、冶山等历史文化街区(风貌区)保护修复。科学合理开发永泰嵩口、长乐琴江等历史文化名镇(村)资源。大力弘扬闽都文化,强化文物保护和非物质文化遗产传承创新。加强文艺精品创作,促进哲学社会科学、新闻出版、广播影视、文学艺术等事业繁荣发展。认真编纂福州市志和年鉴。鼓励发展文化产业,加快中国船政文化城、海峡非物质文化遗产生态园、中国漆文化产业园等建设。积极培育和践行社会主义核心价值观,加强社会公德、职业道德、家庭美德和个人品德教育,提升城乡居民文明素质,支持发展志愿服务。

　　(七)切实保障和改善民生。

　　多渠道促进就业创业。完善就业再就业各项政策和公共就业创业服务体系,做好高校毕业生、农村转移劳动力、就业困难人员和退役军人等重点群体就业援助工作,争取城镇新增就业13.3万人,转移农业富余劳动力4.6万人,城镇失业人员再就业8500人,实施职业技能培训4.3万人次。推行工资集体协商,加强劳动关系三方协调、劳动争议仲裁和劳动保障监察,维护劳动者和企业合法权益,构建和谐劳动关系。

　　全面增强社会保障能力。稳步实施城乡居民社会养老保险一体化,提高城乡居民基础养老金、城乡低保、农村“五保”和城乡居民医保财政补助标准,推进城乡居民基本医疗保险制度整合,完善对被征地、收海农民的多元保障机制。新开工建设1.3万套保障性住房,基本建成1.1万套保障性住房,同时加快解决被拆迁群众逾期安置问题。大力发展养老服务业,着力构建以居家养老为基础、社区服务为依托、机构养老为支撑的养老服务体系。保障妇女权益,关心青少年发展,积极发展社会福利、社会救助、社会慈善和残疾人事业,加快市级社会福利中心、福乐家园等建设。抓好“菜篮子”工程建设,新建蔬菜基地6000亩,保障农产品供应量足、价稳、质优。继续实施“食品放心工程”,坚决治理“餐桌污染”,强化食品生产、流通、销售全流程安全监管,保障广大人民群众食品安全。

　　不断推动社会治理创新。做好村(居)基层自治组织换届选举工作,促进村(居)服务规范化、标准化建设。深化“平安福州”建设,创新立体化社会治安防控体系,严密防范、依法打击各类违法犯罪活动。健全重大决策社会稳定风险评估机制,加大社会矛盾纠纷调处力度,进一步畅通和规范群众诉求表达渠道。加强全民普法,弘扬宪法精神。做好法律服务和法律援助工作。强化道路交通、液化气、校园等重点行业、重点领域、重点部位安全监管和隐患排查治理,坚决防范和遏制重特大事故发生。支持工会、共青团、妇联等人民团体广泛参与社会治理。推进社会组织孵化基地建设,培育发展和规范管理社会组织。发挥市民公约、乡规民约等社会规范在社会治理中的积极作用。提升流动人口服务管理水平,做好社区矫正、刑满释放人员安置帮教和青少年事务社工等工作。大力开展政务诚信、商务诚信、社会诚信建设,着力建设“诚信福州”。积极促进民族乡村经济社会发展,维护宗教领域和谐稳定。扎实做好防震减灾、科普、气象、保密等工作。加快建立军民融合发展机制,进一步提高军转安置和优抚工作水平,加强国防教育、国防动员、国防后备力量建设和海防、人防、反走私等工作,争创双拥模范城八连冠。

　　(八)努力打造人民满意的法治政府和服务型政府。

　　进一步依法认真履职尽责。严格依照法定权限和程序行使权力、履行职责,建立权力清单、责任清单、负面清单等制度。健全公众参与、专家论证、风险评估、合法性审查等科学民主决策制度,不断提高政府科学决策水平。积极倡导“敢为、能为、有为”精神,敢闯敢试、敢于担当、敢于负责,切实做到“想干事、会谋事、敢担事、能成事”。大力弘扬“马上就办”的优良作风,全面提高政府执行力和机关效能、服务质量,坚决纠正不作为、乱作为,坚决克服“庸懒散拖”,着力破除“熟人经济”和本位主义思想。

　　进一步提高依法行政水平。牢固树立法治意识,坚持领导干部带头学法、模范守法,健全行政机关工作人员学法用法制度。完善行政执法程序,细化、量化行政裁量标准,确保严格规范公正文明执法。提高政府立法质量,积极推行政府法律顾问制度。支持司法机关依法独立公正行使职权。依法主动自觉接受人大及其常委会的法律监督、工作监督和政协的民主监督,加强与各民主党派、工商联和各人民团体、社会各界人士的沟通联系,切实提高办理人大代表建议、政协提案的满意率、落实率。大力推行政务公开、政府信息公开,主动接受广大人民群众的监督。

　　进一步强化为民服务意识。巩固拓展党的群众路线教育实践活动成果,深入开展“四个万家”等活动,察民情、知民意、暖民心,不断密切与群众的血肉联系。针对群众关注的热点难点问题,积极为群众办实事、解难题、谋福祉。持续整改“四风”问题,坚决纠正损害群众利益的不正之风,推进公务用车制度改革、“三公”经费公开等工作。认真执行廉政准则,全面加强惩治和预防腐败体系建设,强化行政监察和审计监督,严肃查处各类违法违纪行为,确保人民赋予的权力始终用来为人民谋利益。

　　各位代表,福州发展正面临前所未有的大好机遇,美好的新前景令人鼓舞,人民的新期待催人奋进。让我们紧密团结在以习近平同志为总书记的党中央周围,在省委、省政府和市委的领导下,进一步抢抓机遇、开拓创新、激情创业,为全力推进福州新区开放开发,在更高起点上加快建设闽江口金三角经济圈,加快福州科学发展跨越发展而努力奋斗!
